# Calcified Pilocytic Astrocytomas and Calcifying Pseudoneoplasms of the Neuraxis: A Diagnostic Challenge

**DOI:** 10.7759/cureus.51765

**Published:** 2024-01-06

**Authors:** Rebeca Hernández-Reséndiz, Eliezer Villanueva-Castro, Edgardo de Jesus Mateo-Nouel, Erick Gómez-Apo, Carlos Peñafiel-Salgado, Citlaltepetl Salinas-Lara, Martha Lilia Tena-Suck

**Affiliations:** 1 Department of Pediatric Neurology, Hospital Angeles Universidad, Mexico City, MEX; 2 Department of Neurosurgery, Instituto Nacional de Neurología y Neurocirugía Manuel Velasco Suárez, Mexico City, MEX; 3 Department of Pathology, Hospital General de México, Mexico City, MEX; 4 Department of Neuropathology, Instituto Nacional de Neurología y Neurocirugía Manuel Velasco Suárez, Mexico City, MEX

**Keywords:** psammoma bodies, dystrophic calcification, calcified pilocytic astrocytoma, calcifying pseudoneoplasm of the neuraxis, tumor calcifications, senescence-associated secretory phenotype, glioma, tumor markers, capnon

## Abstract

Pilocytic astrocytoma (PA), recognized as the most prevalent central nervous system (CNS) tumor, has long been associated with calcifications, a characteristic often attributed to benign or indolent growth patterns. In this study, we explored the calcified attributes in these tumors that beckon a deeper understanding.

This is a retrospective study, on a set of seven cases, with a histopathological diagnosis of pilocytic astrocytoma with calcifications and psammoma bodies (PB). Despite an encouraging overall survival outcome, the recurrence in four cases cast some doubt on the conventional classification.

The histological study of these cases revealed a spectrum of calcifications, varying in size and morphology, all of which exhibited positive reactivity to glial fibrillary acidic protein (GFAP), osteoconduction, and osteopontin. Notably, the immunohistochemistry showed hyaline bodies displaying an atypical immune profile, strikingly negative for vimentin and GFAP, and a robust positivity for epidermal growth factor receptors (EGFR), tumor necrosis factor-alpha (TNF-α), and interleukin 1 beta (IL-1β). These results stimulated speculation that the identity of these calcified tumors may have extended and potentially embraced the realm of calcifying pseudoneoplasms of the neuraxis (CAPNON), underscored by intense pilot gliosis.

This study transcends mere anatomical exploration; it delves into the intricacies of calcified tumors, casting a spotlight on the dynamic interplay between PA and CAPNON. As we traverse the frontiers of neuro-oncology, these findings pave the way for innovative avenues in the diagnostics and therapeutics of these tumors.

## Introduction

Pilocytic astrocytoma (PA) constitutes 56% of all gliomas and is a slow-growing glioma, classified as grade I by the World Health Organization (WHO) [1], and it is the most common glial neoplasm in children and is found in the cerebellum [2]. It grows during the first two decades of life; only one-third of the patients are older than 18 years of age, and only 17% are older than 30 years of age. In adults, one-half of the tumors are supratentorial [2]. There are no characteristic clinical features; PAs usually follow a slow course; signs and symptoms are related to the size, location, and presence of associated hydrocephalus, with an extremely high survival rate of over 90% at 10 years of age [3]. The appearance of PA on brain magnetic resonance imaging (MRI) is variable and depends on the tumor's size and structure [4]. T2-weighted MRI sequences can show isointense and hyperintense lesions containing features suggestive of calcification and mildly contrast-enhancing cysts of variable sizes [5,6]. Gross surgical resection is curative in most patients [7,8]. Although others indicate that PA may show aggressive behavior macroscopically, PAs typically appear as well-circumscribed, cyst-like tumors with a discrete mural nodule [1].

Histologically, PA frequently exhibits a biphasic pattern, with more solid areas composed of bipolar areas and brightly eosinophilic Rosenthal fibers (RFs) alternated with looser, softer areas containing microcysts and eosinophilic granular bodies (EGBs) [1,3]. Pleomorphism, cellular atypia, mitotic features, avascular hyalinization, and dystrophic calcification (DC) are infrequent; however, vascular proliferation may be found, accompanied by a hyaline vascular wall as well [4]. In very few cases, a possible transformation to a more malignant astrocytic tumor has been observed in association with mutations in the isocitrate dehydrogenase (IDH) mutant or wild-type genes, including the canonical *R132H* type [1]. It has been shown that IDH mutations are absent in PAs and present in association with *KIAA1549-*v-Raf murine sarcoma viral oncogene homolog B (*BRAF*) gene fusion, mitogen-activated protein kinase (MAPK), and mammalian target of rapamycin (mTOR) activation pathways [1], which result from a somatic chromosomal duplication of 7q34 [9], an indicative tool to differentiate it from diffuse astrocytoma [5]. Fibroblast growth factor receptor 1 (FGFR1) and the Tyrosine-protein phosphatase non-receptor type 11 (*PTPN11*) gene are localized in 12q24.1. PA has been linked to this chromosome, which encodes tyrosine phosphatase Shp2, novel neurotrophic receptor tyrosine kinase 2 (NTRK2) rearrangement, and MAPK pathway fusion genes. Oligodendrocyte transcription factor 2 (OLIG2) expression is restricted to pseudo-oligodendroglial cells [1]. While calcification is more commonly regarded as a feature of benign or slow-growing tumors, the detailed original mechanism remains unclear [5]. A few cases of calcified pilocytic astrocytoma (CPA) have been published as case reports [10-12]. Although calcification is not common, it has been reported more frequently in the optic nerve, hypothalamus, and thalamus, as well as in superficially located cerebellar tumors. Massive calcification is not a common feature in PAs and can lead to difficulties in radiological and pathological differential diagnoses [12].

Calcifying pseudoneoplasms of the neuraxis (CAPNON) is a non-neoplastic lesion with fewer than 100 documented cases. At 40%, it can be localized intraspinally and at 60% intracranially. It might resemble other calcifying intra-axial lesions, and their symptoms depend on their localization [13].

The aim of this paper is to report seven cases of calcified pilocytic astrocytoma in adults with densely dystrophic calcification and psammoma body (PB) formations. This is a clinicopathological and immunohistochemical approach.

## Materials and methods

Patients and study design

We carried out a retrospective study on a set of seven cases chosen from a larger cohort of 25 patients who had been diagnosed with pilocytic astrocytoma (PA) and had undergone surgical intervention within five years at the Manuel Velasco Suárez National Institute of Neurology and Neurosurgery in Mexico City.

The study's criteria for inclusion encompassed the following: 1) individuals aged 15 years and older, 2) brain tumor surgery in our hospital, 3) histopathological validation for pilocytic astrocytomas, and 4) the presence of calcifications and psammoma bodies (PB). The study's criteria for exclusion are the following: 1) an incomplete clinical record and 2) unavailable histopathological staining resources.

Methodology

Samples and tissues were obtained during the surgical resection of the tumors. The cytopathological, histopathology, and immunohistochemistry analysis was conducted by a group of four neuropathologists.

Tissue sections were cut at 4 μm and deparaffinized in xylene and absolute alcohol using standard immunohistochemistry techniques as previously described. Endogenous peroxidase activities were blocked with a solution of 3% hydrogen peroxide and absolute alcohol 1:1 for 15 minutes. Heat-induced antigen retrieval was performed with a pressure cooker (122°C±2°C) for 45 seconds at 15±5 PSI in citrate buffer (pH 6.0). Immunohistochemistry was performed using the different primary antibodies used at a dilution of 1:100 incubated for 45 minutes at room temperature, followed by labeled polymer-horseradish peroxidase (HRP) anti-rabbit immunoglobulin G (IgG) incubated for 30 minutes, and visualized with 3,3′-diaminobenzidine (DAB) (brown in color). The slides were counterstained with hematoxylin using the Diagnostic BioSystems KP50 and KP500 (Pleasanton, CA). Images were acquired using a ZEISS Axiolab microscope (Oberkochen, Germany) and a ZEISS digital camera (Oberkochen, Germany). Subsequent steps were performed with a commercial kit and according to the manufacturer's instructions. For negative controls, the primary antibody was replaced by unspecific immunoglobulin G from nonimmunized mice or rabbits, as appropriate. Additionally, the intensity of the staining was assessed semiquantitatively (0-3), and the staining pattern (membranous, cytoplasmic, and nuclear) was determined.

The primary antibodies that were searched for in the tissue samples were the following: isocitrate dehydrogenase (IDH) 1 (GTX133076, GeneTex; dilution: 1:100), isocitrate dehydrogenase (IDH) 2 (GTX103682, GeneTex; dilution: 1:100), osteocalcin (sc-30044, Santa Cruz; dilution: 1:100), osteonectin (OP) (AM387-5M, BioGenex; dilution: 1:100), Ki67 (MU410-UC, BioGenex; dilution: 1:100), neuronal nuclei (NeuN) (Z21178, ZETA; dilution: 1:100), epidermal growth factor receptor (EGFR) (Pu335-UP, BioGenex; dilution: 1:100), basic fibroblast growth factor (bFGF) (AF-213-NA, Biothene research and development (R&D) system; dilution: 1:100), N-methyl-D-aspartate (NMDA) (cs17822, Santa Cruz; dilution: 1:100), derivative plaqueless growth factor (DPGF) (FMU376-UP, BioGenex; dilution: 1:100), vimentin (MU074-UP, BioGenex; dilution: 1:100), tumor necrosis factor-alpha (TNF-α) (anti-TNF-α antibody, ab9635, Abcam; dilution: 1:100), tumor necrosis factor-gamma (TNF-γ, CC302, AbD Serotec; dilution: 1:100), glial fibrillary acidic protein (GFAP) (MU020-UC, BioGenex; dilution: 1:100), nestin (anti-nestin antibody {10C2}, ab22035, Abcam; dilution: 1:100), and hypoxia-inducible factor 1-alpha (HIF-1α {28b}, sc-13515, mouse IgG1, 329-530, Santa Cruz; dilution: 1:100).

The differentiation of primary antibodies used was assessed on a semiquantitative scale: no staining (-), focal <10% of the cells (+); 10-50% of cells or weak staining in >50% of cells (++), and strong staining of >50% of cells (+++). The Molecular Immunology Borstel 1 (MIB-1) index was used for Ki67, and the microvascular density (MVD) labeling index was used for cluster of differentiation 34 (CD34).

Statistical analysis

Vital demographic and clinical parameters were methodically collected from the encompassing population. It is noteworthy that the evaluation of intraoperative cytopathology was undertaken in a select subset of three cases, guided by the surgical team's discretion, and this endeavor was further complemented by a comprehensive histopathological evaluation of all seven tumors. The entire cohort underwent an exhaustive process of immunohistochemical analysis and immunoreactivity assessment.

The results were analyzed by descriptive statistics and are presented as measures of central tendency. Continuous variables were represented through the utilization of mean values, while categorical variables were effectively elucidated in terms of their respective percentages.

## Results

Our sample included two females (28.6%) and five males (71.4%). The main age was 38.28 years (range: 15-67).

The anatomical localization of the CAPNON lesions displayed three cases (42.85%) situated in the supratentorial region and four cases (57.15%) within the infratentorial territory. The morphological dimensions exhibited an average tumor diameter size of 39.6 mm, spanning from 34 mm to 46 mm, emphasizing their substantial physical presence within the neuroanatomical landscape.

Of significance, a subset of patients (28.6%) had a familial history of central nervous system (CNS) tumors, potentially indicating an underlying genetic predisposition. The clinical course was marked by an average symptom progression period of eight months, further delineating the temporal aspects of the disease trajectory.

A complete tumor resection was executed in five patients (71.4%) and partial resection in the rest (28.6%). The adjunctive inclusion of adjuvant radiotherapy was embraced by five patients (71.4%), as a strategic measure to fortify treatment efficacy.

The recurrence of the CAPNON occurred in four cases (57.14%), necessitating comprehensive clinical investigation.

This clinical inquiry concluded with the clear transition of four patients (57.14%), reaffirming the pathological dynamics. Table 1 shows the clinical characteristics. This study offers a multidimensional portrayal of CAPNON, their clinical profiles, therapeutic encounters, and disease trajectories, thereby contributing to the broader understanding of these neuroepithelial entities.

**Table 1:** Clinical characteristics ST, supratentorial; IT, infratentorial

Clinical data	Case 1	Case 2	Case 3	Case 4	Case 5	Case 6	Case 7
Gender	Female	Female	Male	Male	Male	Male	Male
Age	15	33	25	38	55	67	35
Localization	ST	ST	IT	IT	IT	IT	ST
Tumor size diameter	35 mm	34 mm	40 mm	39 mm	45 mm	46 mm	38 mm
Family history	Yes	No	No	No	No	No	Yes
Symptom evolution time	4 months	6 months	6 months	12 months	7 months	8 months	13 months
Extend of resection	Total	Total	Total	Partial	Partial	Total	Total
Radiotherapy	Yes	No	No	Yes	Yes	Yes	Yes
Recurrence	Yes	No	No	Yes	Yes	Yes	No
Follow-up in months	12	17	12	24	32	30	14
Deceased	No	Yes	No	Yes	Yes	Yes	No

Cytopathology

**Figure 1:**
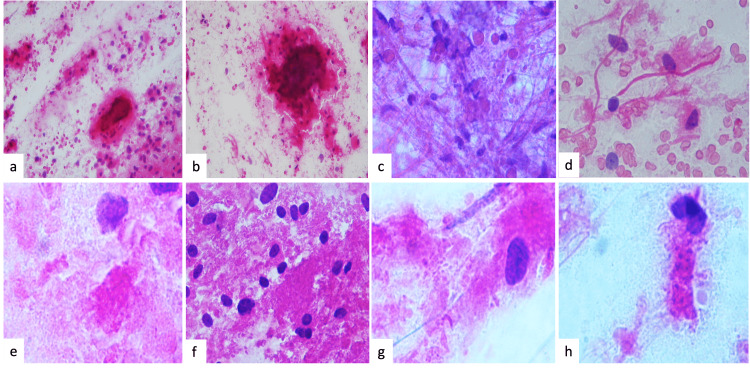
Cytopathology of tumor samples Intraoperative hematoxylin and eosin (H&E) smears. (a and b) The background was dirty, and at low magnification, eosinophilic and basophilic amorphous formations that correspond to calcifications were observed (H&E: ×200). (c) Fibrillar appearance and eosinophilic bodies in (d) the Rosenthal fibers were observed (H&E: ×200). (e) In a close-up of the background, amorphous granules of varying sizes and granular eosinophilic granular bodies are shown. (f) Immersed bipolar astrocytes in a densely granular background, (g) some gemistocytic astrocytes with granular cytoplasm, and (h) some astrocytes with varying degrees of granulations and cytoplasmic mineralization are shown (H&E: ×400)

Cytopathological stains were performed in only three patients intraoperatively, in which varying degrees of calcification were observed due to a suspected diagnosis. In the rest, it was not necessary because the histopathological results would not change the surgical plan of maximum safe resection. The background was dirty, and at low magnification, eosinophilic and basophilic amorphous formations that correspond to calcifications were found (Figure 1a, 1b). Fibrillar appearance, eosinophilic bodies (Figure 1c), and the Rosenthal fibers (Figure 1d) were also observed. In a close-up of the background, amorphous granules of varying sizes and eosinophilic granular bodies are shown (Figure 1e). Bipolar astrocytes in a densely granular background (Figure 1f), astrocytes of gemistocytic appearance with granular cytoplasm (Figure 1g), and some astrocytes showing varying degrees of cytoplasmic granulations and mineralization are also visible (Figure 1h).

Histopathology

**Figure 2:**
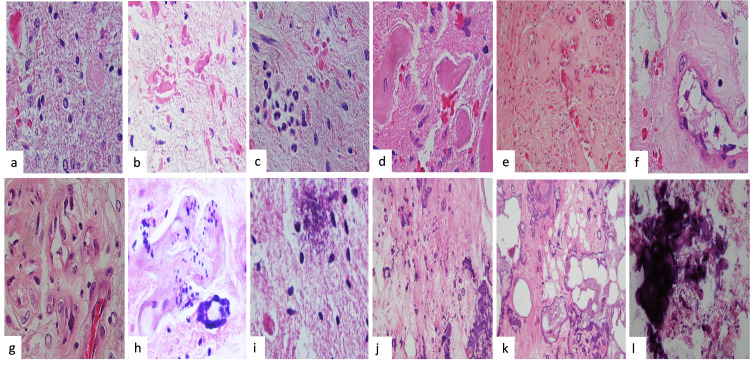
Histopathology Histological features of the tumors: (a) stromal rarefaction, with microcystic appearance and granular bodies; (b) Rosenthal fiber formation; (c) inflammation; and (d-f) vascular hyalinization. (g) Some vessels showed thickening with different thicknesses and occlusion of light and endothelial hyperplasia; some endothelial cells showed focal atypia (H&E: ×400). (g) Dystrophic calcifications of various shapes and sizes were observed. (h) Rosary-shaped calcifications were observed in the periphery of blood vessels. (h) We observed calcium deposits and mineralization in the stroma in varying ways and (i) variable sizes of calcifications from empty-looking bodies to eosinophilic bodies such as starchy bodies, (j) with psammoma bodies and (k and l) with amorphous nodular versus granular microcalcifications with an appearance of basophilic material deposition (H&E: ×200) H&E: hematoxylin and eosin

Histopathological results are shown in Table 2. Patients showed focal changes of pilocytic astrocytoma with stromal rarefaction, microcystic appearance, and granular bodies (Figure 2a). The Rosenthal fibers (Figure 2b), inflammation foci (Figure 2c), vascular hyalinization (Figure 2d), thickened vessels (Figure 2d, 2e), and the glomeruloid-like appearance of the hyalinized vessels (Figure 2f) were seen. Calcifications showed variable form and sizes, with calcifications located in the periphery of blood vessels (Figure 2g), calcium deposits and mineralization in the stroma (Figure 2h), varying sizes of calcifications from empty-looking bodies (Figure 2i) to psammoma bodies (Figure 2j), and amorphous nodular versus granular microcalcifications (Figure 2k, 2l).

**Table 2:** Histopathology findings +, mild presence (<33% of the microscope field of view); ++, moderate presence (33%-66% of the microscope field of view); +++, severe presence (>66% of the microscope field of view) VDC: vascular dystrophic calcifications

Histopathology	Case 1	Case 2	Case 3	Case 4	Case 5	Case 6	Case 7
Cellular atypia	+	No	No	No	No	No	+
Bipolar astrocyte	++	+	+	+	+	+	++
Pleomorphism	+	+	No	No	No	No	+
Gemistocytes	+	No	No	No	No	No	+
Rosenthal fibers	++	++	++	++	++	+	+++
Granular bodies	++	+	+	+	+	+	+
Necrosis	+	+	+	No	No	No	No
Dystrophic calcifications (%)	1	2	3	3	3	3	2
Psammoma bodies (%)	++	++	+	+++	+++	++	+
VDC	No	Yes	Yes	Yes	Yes	Yes	No
Vascular hyalinization	++	+	++	++	+++	++	+
Mineralized stroma	+	++	++	+++	+++	++++	+
Avascular areas	No	+	+	++	++	+	No
Microcysts	Yes	Yes	No	No	No	No	Yes

Immunohistochemistry

**Figure 3:**
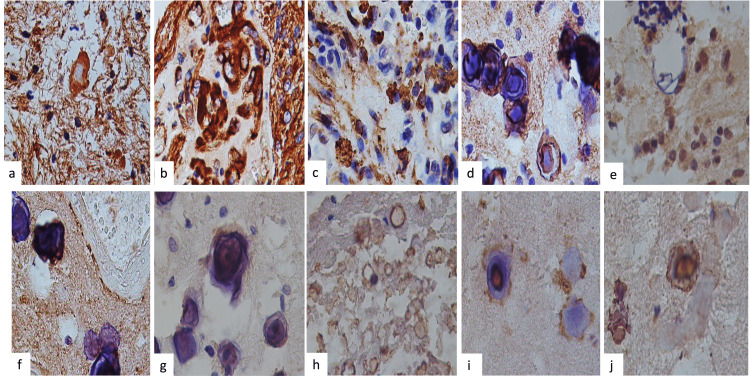
Immunohistochemistry (IHC) Immunohistochemistry features: (a) stromal cells and neutrophils were vimentin-positive, and the Rosenthal fibers (RFs) were positive only in the peripheral region (b). We observed vimentin positivity in the blood vessel wall and endothelial cells. (c) GFAP was positive in the stroma cells in all cases, both in normal and bipolar astrocytes and in gemistocytes, in variable forms of immunoexpression in the RFs and gemistocytes, and in dense structures that we attribute to RFs and (d) a variable positive immunoreaction, particularly at the PB periphery. (e) Vimentin has weak or mild immunoexpression in the blood vessel wall. (f) Nestin was only intensely positive in bipolar astrocytes. (g) Osteonectin was slightly positive in the PB outer wall, (h) and osteopontin was more intense in the walls of empty calcifications, (i) hyaline bodies, and (j) RFs (IHC original magnification: ×400) GFAP, glial fibrillary acidic protein; PB, psammoma body

**Figure 4:**
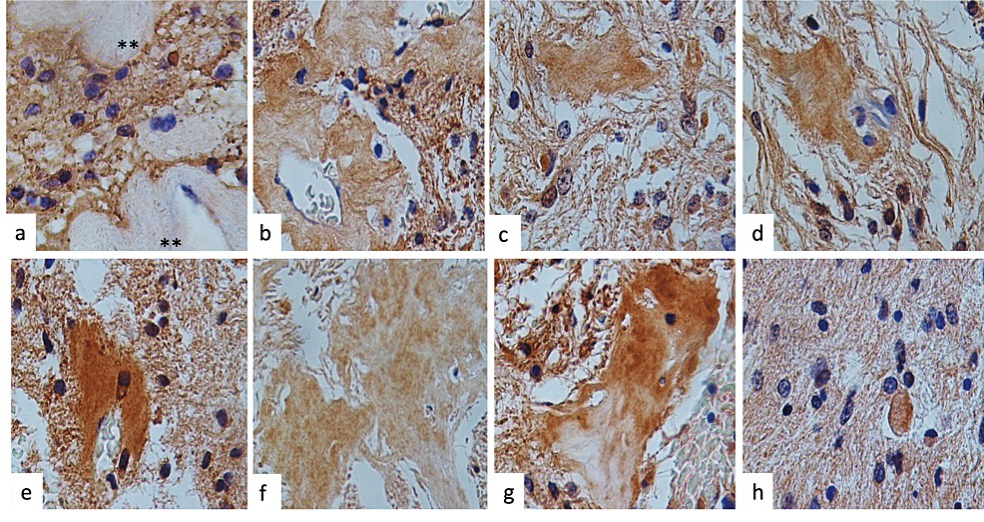
Immunohistochemistry and immunoreactions (a) Negative reactions included GFAP hyaline bodies and hyaline vascular wall. Hyaline bodies were positive for immunoreaction to (b) bFGF, (c) EGFR, (d) DPGF, (e) TNF-α, (f) MDA, and (g) HIF-1 and (h) observed RF-positive for immunoreaction to MDA (original magnification: ×400) GFAP, glial fibrillary acidic protein; bFGF, basic fibroblast growth factor; EGFR, epidermal growth factor receptor; DPGF, derivative plaqueless growth factor; TNF-α, tumor necrosis factor-alpha; MDA, malondialdehyde; HIF-1, hypoxia-inducible factor 1; RF, Rosenthal fiber

In Table 3, we show the immunohistochemistry results according to each primary antibody used. All tumors were positive for GFAP (Figure 3a) and vimentin (Figure 3b). Vimentin was positive in the stroma, the neuropil, the peripheral portion of the RF, the wall of the blood vessels, and the endothelial cells (Figure 3b). Hyaline bodies were positive for immunoreaction to bFGF, EGFR, DPGF, TNF-α, malondialdehyde (MDA), and HIF-1 and observed RF-positive for immunoreaction to MDA (Figure 4). Dystrophic calcifications and psammoma bodies were negative for bFGF, EGFR, PDGF, TNF-α, MDA, and HIF-1. The expression of IDH1, IDH2, and neuronal nuclei (NeuN) was negative in all cases.

**Table 3:** Immunohistochemistry No staining (-), focal <10% of the cells (+), 10%-50% of cells or weak staining in >50% of cells (++), and strong staining of >50% of cells (+++) GPAF, glial fibrillary acidic protein; MVD, microvascular density; RFs, Rosenthal fibers; GB, granular bodies; OP, osteopontin; ONT, osteoconduction; bFGF, basic fibroblast growth factor; TNF-α, tumor necrosis factor-alpha; MDA, malondialdehyde; HIF-1a, hypoxia-inducible factor 1-alpha; EGFR, epidermal growth factor receptor; NeuN, neuronal nuclei; IDH1/2, isocitrate dehydrogenase 1 and 2; CD34, cluster of differentiation 34

Antibody used	Case 1	Case 2	Case 3	Case 4	Case 5	Case 6	Case 7
GFAP	+	++	+	+	+	+	++
Ki67	2%	3%	2%	1%	1%	1%	3%
MVD (CD34)	25%	28%	10%	10%	10%	9%	30%
Vimentin	+	++	+	+	+	+	++
GFAP+RFs	+	+	+	+	+	+	+
GFAP+GB	+	+	++	++	++	++	+
Nestin	++	++	+	+	+	+	++
OP	+	++	++	+++	+++	+++	++
ONT	+	++	++	+++	+++	+++	+
bFGF	+	+	+	+++	+++	+++	+
TNF-α	++	+	+	+	+	+	++
MDA	+	+	+	+++	+++	+++	+
HIF-1α	+	+	+	++	+++	+++	+
EGFR	+	+	+	+	+	+	+
NeuN	-	-	-	-	-	-	-
IDH1/2	-	-	-	-	-	-	-

## Discussion

In the present study, we present seven cases of PA with abundant calcifications and PBs that were diagnosed as CPA. The important matter is to differentiate CAPNON from CPA because the prognosis may be different; however, despite the radiotherapy they were given, some of the cases relapsed and even presented greater calcifications.

Dystrophic calcification (DC) is not a common finding, neither in PA nor in other tumors, and has been reported more frequently in the optic nerve, hypothalamus/thalamus, and superficially located cerebral tumors. Only a few cases have been reported [1], and familial and genetic brain calcifications are associated with primary familial brain calcification (PFBC) gene mutations [6]. Calcification may be due to idiopathic hypoparathyroidism as a differential diagnosis [1].

Calcium deposits take a long time to form, which explains why calcifications appear more frequently in slow-growing glial tumors. The rate of calcification in glial tumors ranges from 9.3% to 19%, and up to 25% of PAs have calcification, though only four cases of extensive massive calcification associated with PA have been reported [1,10]. Eosinophilic granular bodies (EGBs) from globular aggregates with astrocyte processes and the granular material that calcifies and mineralizes can be found within these tumors. EGBs are usually periodic acid-Schiff positive (PAS+) and react to alpha-antichymotrypsin or alpha-1 antitrypsin [1]. In our study, we found that these EGBs were immunoreactive to GFAP, bFGF, PDFG, EGFR, TNF-α, MDA, and HIF-1α, features corresponding to degenerative changes of both astrocyte fragments and cell detritus. These could be the same as the hyalinized vessel walls resulting from paracrine secretion and/or destruction, as well as the degenerative changes of the vascular wall. These degenerative changes are observed in the smear of the intraoperative study as a cumulus of secreted material that forms structures after calcification. The hyalinization of the blood vessels is another feature of PAs. The microvascular proliferation of PAs accounts for the contrast enhancement that accompanies these tumors on cross-sectional imaging [14].

PBs are concentric lamella-calcified structures, said to represent a process of DC. They are mostly in the form of extracellular hyaline globules bounded by neoplastic cells or, in a smaller number of cases, intracytoplasmic bodies from intact tumor cells. However, cellular degeneration and necrosis, which lead to the loss of neoplastic cells, may be the underlying cause [14]. Based on the above findings, it is proposed that rather than being the outcome of DC of dead or dying tissue, PBs may indeed mean an active biologic process that ultimately leads to the death of tumor cells or the retardation of the growth of the neoplasm. DCs and PBs may also assist as a barrier against the spread of neoplasms [11].

Notwithstanding plentiful ancillary studies over a span of three and a half decades, the formation of PBs and DCs remains a poorly understood mechanism. Oligodendroglial-like cells, hyperchromatic cells, pleomorphism, glomeruloid vascular proliferation, and leptomeningeal infiltration may be found, but they do not indicate malignancy in PA. Furthermore, PA expresses alkaline phosphatase in degenerative tissues, leading to pathological mineralization [12,15]. Normally, calcified brain tumors comprise oligodendrogliomas, low-grade astrocytomas, craniopharyngiomas, meningiomas, pineal gland tumors, ependymomas, and intraventricular tumors. Choroid plexus tumors, central neurocytomas, metastatic tumors, and those in the degenerative or necrotic tissue have been associated with calcifications [12].

Calcifying pseudoneoplasms of the neuraxis are rare and typically benign lesions that can arise anywhere within the CNS [16]. On radiographic examination, CAPNON presents as centrally calcifying lesions, and histopathological analysis usually shows a foreign body reaction with giant cells, ossification, and the formation of PB, extensively calcified lesions mixed with reactive astrocytosis. CAPNON stains positively for GFAP, epithelial membrane antigen (EMA), and vimentin, a subunit of an intermediate filament of a structural protein that confirms the mesenchymal origin of tumors called vimentin [17] but is frequently negative for the S100 protein [16]. Fibro-osseous components that seem to characterize an atypical type of bone metaplasia and emulate neuroglial tumors such as astrocytomas with calcification, oligodendrogliomas, or hamartomas should also be included in the differential diagnosis [18].

CAPNON should draw attention to the differential diagnosis of calcified lesions; an inaccurate diagnosis can result in unnecessary therapies. The tumor has the features of a foreign body reaction, with giant cells, ossification, and the development of PBs. On imaging, they can easily be confused with malignant lesions such as chondrosarcoma, chondroblastoma, or even more benign pathologies such as meningioma [18].

Senescent cells activate genetic programs that not only irreversibly inhibit cellular proliferation but also endow these cells with distinctive metabolic and signaling phenotypes, including cancer. These cells specialize in the secretion of a vast array of pro-inflammatory cytokines, chemokines, and growth factors, collectively known as the senescence-associated secretory phenotype (SASP), by autocrine and paracrine pathways that can affect neighboring cells. Secretory factors not only play a positive role in driving antitumor immunity but also exert negative influences on the microenvironment [19].

In sporadic PA, focal chromosomal gains have been found on chromosome 7q34 (53%-88%), in the region of the v-Raf murine sarcoma viral oncogene homolog B (*BRAF*), which is highly specific in PA. *BRAF* activation promotes clonogenic growth in neural progenitor cells [8]. Neurofilament light chain (NFL) protein is found in the core of CAPNON lesions [20].

Therapy-induced senescence is a major cellular response to chemotherapy in solid tumors. While calcification is more commonly regarded as a feature of benign or slow-growing tumors, the detailed mechanism remains unclear [21]. In vitro and in vivo studies suggest that *BRAF* and MAPK/extracellular-signal-regulated kinase (ERK) (MEK) inhibitors may be potentially effective therapies for some molecular subtypes of PA [8]. Due to the presence of NFL in CAPNON, it is believed that therapies directed against NFL could have a therapeutic effect on these tumors [20]. Further studies are needed.

The formation of eosinophilic amorphous structures or hyaline bodies, the hyaline vascular wall that is peripherally reinforced by the astrocyte response, and the presence of fine calcium deposits and variable calcifications all contribute to the formation of psammoma bodies in this tumor [11]. Concentric bodies with variable expression of GFAP, bFGF, PDGF, EGFR, TNF-α, MDA, and HIF-1α were found, so we suggest that hypoxic changes, as well as oxidative stress activity, an inflammatory process, cytokine secretion, growth factors, and SASP, may be underlying this phenotype. They are involved in the mechanism of the formation of these calcifications, and radiotherapy may trigger this mechanism [19]. We suggest that this process can be slow and progressive until complete calcification or the formation of stones, which often allows surgery and is usually curable [8].

The tumor marker description in CAPNON may be useful for much more than its classification. For example, the standard in the treatment of high-grade gliomas is the maximal surgical removal of the tumor and medical treatment with temozolomide. Resistance to this chemotherapeutic agent has led to the search for a targeted therapy to attack various molecular entities and thus offers an advantage in the effectiveness of the treatment and reduction of collateral damage. Current molecularly targeted therapies for glioblastoma are directed at EGFR, mTOR, vascular endothelial growth factor (VEGF), MEK, phosphoinositide 3-kinase (PI3K), and *BRAF*. Combined therapies, or monotherapies, can be used [22], and this suggests possible new pads for CAPNON treatment.

PAs have a specific feature of benign neoplasm that in many cases shows histological features that are normally seen in higher-grade neoplasms and appear inappropriate for a slow-growing brain tumor with fairly bland histological characteristics, such as microvascular proliferation, intratumoral hemorrhage, the infiltration of surrounding tissues and structures, or leptomeningeal dissemination. In those cases, an incomplete surgical procedure is related to a bad prognosis [23]. However, the hemorrhagic presentation of a calcified PA is extremely uncommon. Endothelial proliferation may be the main cause of bleeding in these lesions. Histopathological findings may reveal a hypervascular appearance and a hyaline vascular wall pattern. When calcifications are present within some micro-hemorrhagic consequences, a PA diagnosis should be considered [24].

The limitation of this study is the design because it was performed retrospectively and data recollection was difficult. Also, the small sample size in just one center can have a poor effect on the population. The methodology must be carried out by specialized pathologists and with specific staining methods so it can represent a difficulty in its replicability.

## Conclusions

It is very important to discuss the relevant differential diagnoses of pilocytic astrocytoma in each tumor location. PA can present aggressive behavior with an atypical location and early recurrence in adult patients, such as the seven patients in this report. In the seven patients in this report, the hyaline bodies were immunoreactively positive for bFGF, EGFR, DPGF, TNF-α, MDA, and HIF-1. The Rosenthal fibers were positive for HIF-1 and MDA. Hyaline bodies and the Rosenthal fibers are associated with oxidative stress and hypoxic changes. We suggest that radiotherapy can be a contributing factor in the secretion of substances such as cytokines, chemokine factors, and growth factors, as well as the expression of MDA and HIF-1α that participate in oxidative stress and hypoxic mechanisms that cause the appearance of dystrophic calcifications. Cellular senescence must be considered a potent tumor suppressor mechanism. CAPNON and CPA must be distinguished due to their distinct prognoses.
